# Survival analysis via nomogram of surgical patients with malignant pleural mesothelioma in the Surveillance, Epidemiology, and End Results database

**DOI:** 10.1111/1759-7714.13063

**Published:** 2019-04-05

**Authors:** Minglei Zhuo, Qiwen Zheng, Yujia Chi, Bo Jia, Jun Zhao, Meina WU, Tongtong AN, Yuyan Wang, Jianjie LI, Xinghui Zhao, Xue Yang, Jia Zhong, Hanxiao Chen, Zhi Dong, Jingjing Wang, Xiaoyu Zhai, Ziping Wang

**Affiliations:** ^1^ Key Laboratory of Carcinogenesis and Translational Research (Ministry of Education/Beijing), Department of Thoracic Medical Oncology Peking University Cancer Hospital & Institute Beijing China; ^2^ Department of Epidemiology and Biostatistics, School of Public Health Peking University Beijing China

**Keywords:** Malignant pleural mesothelioma, nomogram, SEER, surgery, survival

## Abstract

**Background:**

Malignant pleural mesothelioma (MPM) is a rare but aggressive tumor that originates from the pleura and has a poor prognosis. Eligible patients can benefit from surgery, but their survival is affected by many factors. Therefore, we created a graphic model that could predict the prognosis of surgically treated patients.

**Methods:**

We retrospectively analyzed data from the Surveillance, Epidemiology, and End Results database from 2004 to 2014 to identify the key factors affecting the prognosis of surgically treated MPM patients. On this basis we built a nomogram to predict survival. We then evaluated the performance of the nomogram in a validation cohort.

**Results:**

In a training cohort of 828 cases, independent prognostic factors, including age, gender, histological type, differentiation, N stage, chemotherapy, type of surgery, and lymph node dissection, were identified. We then developed a nomogram to evaluate individual patient survival. In Kaplan–Meier analysis, a higher score in the nomogram was associated with a worse prognosis. We also used a validation cohort consisting of 312 patients to evaluate the performance of the nomogram, which was well calibrated and had good discrimination ability, with concordance indices of 0.715 and 0.656 for the training and validation cohorts, respectively.

**Conclusion:**

This study has improved our understanding of resected MPM and shown that key factors, including age and histological type, are associated with overall survival. The nomogram is a reliable tool that can help clinicians turn individualized prediction into reality and maximize patient benefit by identifying the most beneficial treatment approach.

## Introduction

Malignant pleural mesothelioma (MPM) is a rare tumor that originates in the pleura; it is very aggressive and generally has a poor prognosis.^1^, ^2^, ^3^ Surgery can be beneficial in patients who are healthy enough to tolerate it,^4^, ^5^, ^6^, ^7^ but postoperative survival varies, as many other factors also play a role. Previous studies have found that favorable prognostic factors include a lower age, epithelioid histology, good differentiation, negative lymph node status, and chemotherapy.^4^, ^5^, ^6^, ^7^ However, quantitative data based on large cohorts are lacking.

Recently Wang *et al.* built a nomogram based on data from the United States National Cancer Institute Surveillance, Epidemiology, and End Results (SEER) database to predict the prognosis of patients with MPM,^8^ but this effort was complicated by the fact that the outcomes of patients treated with and without surgery differed significantly.^9^, ^10^, ^11^ In selected patients, surgery can lead to significantly better survival compared to non‐surgical therapy (18 vs. 12 months).^12^ Moreover, there are also some factors that specifically affect the survival of surgically treated patients, such as the type of surgery and postsurgical N status.^10^, ^13^ It is therefore necessary to study the prognosis of the two groups of MPM patients (surgery and non‐surgery) separately.

In this study, we analyzed the prognoses of MPM patients listed in the SEER database who had undergone surgery between 2004 and 2014. Our aim was to identify the key factors affecting prognosis and to use a population‐based database to develop a graphic tool that could enable clinicians to evaluate overall survival (OS), thus facilitating both individualized patient care and clinical research.

## Methods

### Study participants

Patients were selected using International Classification of Diseases for Oncology (ICD‐O)‐3 morphology codes 9050–9053. Patients aged > 18 years who had undergone surgery and were diagnosed with malignant mesothelioma between 2004 and 2014 were included. Surgical information was collected based on the SEER surgery code. The surgical procedures included palliative, radical, and not otherwise specified (NOS). We classified patients into groups based on site‐specific surgery using the primary site codes. Radical surgery was defined as radical resection (code 60); and palliative surgery as local tumor destruction (code 10), local tumor excision (code 20), simple/partial surgical removal of primary site (code 30), enucleation (code 40), and debulking (code 50). Patients who underwent surgery of an unknown type (code 90) were placed in the NOS surgery group.

**Figure 1:**
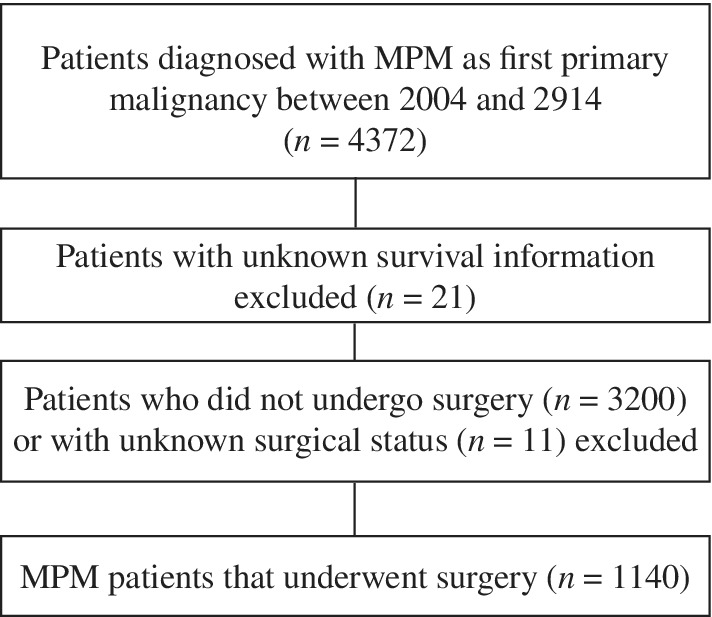
Flowchart showing the selection of study patients. MPM, malignant pleural mesothelioma.

A total of 4372 patients were identified using the code C38.4‐Pleura, NOS in the SEER database. Twenty‐one patients were excluded because no survival data had been recorded. We further excluded 3211 patients with SEER surgery codes indicating that either no cancer‐directed surgery was performed or it was unknown whether cancer‐directed surgery was performed. Finally, 1140 patients who had undergone surgery were enrolled (Fig 1).

The study was exempted from ethical review by the Beijing Cancer Hospital. We obtained the data agreement and downloaded the files directly from the SEER website in accordance with SEER requirements.

### Statistical analysis

We used frequency tabulation and standard descriptive statistics to summarize all patient data. Medians and ranges were recorded as continuous variables, while frequencies and proportions were recorded for categorical variables.

Based on the data of 828 patients who were diagnosed between 2004 and 2011, we constructed a nomogram and then validated it using the data of 312 patients diagnosed between 2012 and 2014. We evaluated the clinicopathologic, demographic, and treatment data on each patient and examined the linearity assumption over continuous variables and proportional hazards (PH) using restricted cubic splines.^14^, ^15^ We transformed continuous variables into proper forms for fitting linearity and PH assumptions. We used log‐log survival plots for categorical variables to identify the PH assumption, and all variables were fitted to the PH assumption. Variables were entered into a multivariate Cox proportional hazards regression model using backward stepwise selection with the Akaike information criterion (AIC), and coefficients of the predictors were calculated. Hazard ratios (HRs) and 95% confidence intervals (CIs) were estimated.^16^ We then constructed the nomogram using the identified prognostic factors to predict one‐year and three‐year survival rates.

The performance of the nomogram, including its discrimination and calibration, was tested using the validation cohort. A model's ability to separate subject outcomes is defined as discrimination and can be quantified by the Harrell C‐index^17^ while the comparison of actual and predicted survival is known as calibration, which can be measured with calibration plots. We used the validation cohort to compare the nomogram‐predicted probability of OS with the observed OS at one and three years. In a well‐calibrated model, the predictions should fall on a 45‐degree diagonal line. In addition, model performance was further evaluated by plotting Kaplan–Meier curves over the quartiles of prediction by nomogram.

We used R software version 3.3.3 for all statistical analyses. The nomogram was developed and modeled using rms of the R package. Reported significance levels were two‐sided, and *P* < 0.05 was taken to indicate statistical significance.

## Results

### Demographic, clinicopathologic, and treatment characteristics

The demographic, clinicopathologic, and treatment characteristics of the training (*n* = 828) and validation (*n* = 312) cohorts are shown in Table 1.

**Table 1:** Demographic, clinicopathologic, and treatment characteristics of study patients

	Training cohort (*n* = 828)		Validation cohort (*n* = 312)	
Characteristics	No. of patients	%	No. of patients	%
Age, years				
Median	67		70
Range	26–95		32–94
Gender				
Male	647	78.1	234	75.0
Female	181	21.9	78	25.0
Race				
White	767	92.6	288	92.3
Black	31	3.7	13	4.2
Other	30	3.6	11	3.5
Histology				
Sarcomatoid	226	27.3	63	20.2
Fibrous	80	9.7	27	8.7
Epithelioid	412	49.8	165	52.9
Biphasic	110	13.3	58	18.6
Differentiation				
Well or moderately	28	3.4	7	2.2
Poorly	49	5.9	18	5.8
Undifferentiated	18	2.2	5	1.6
NOS	733	88.5	282	90.4
Chemotherapy				
Yes	491	59.3	195	62.5
No	337	40.7	117	37.5
Radiotherapy				
Yes	222	26.8	63	20.2
No	606	73.2	249	79.8
Primary tumor location				
Bilateral	15	1.8	3	1.0
Left‐sided	313	37.8	109	34.9
Right‐sided	500	60.4	200	64.1
Clinical stage				
I	128	15.5	59	18.9
II	151	18.2	53	17.0
III	267	32.2	91	29.2
IV	282	34.1	109	34.9
N stage				
N0	540	65.2	201	64.4
N1	111	13.4	100	32.1
N2	142	17.1	4	1.3
N3	11	1.3	2	0.6
NX	24	2.9	5	1.6
T stage				
T1	164	19.8	73	23.4
T2	218	26.3	90	28.8
T3	220	26.6	67	21.5
T4	222	26.8	79	25.3
TX	4	0.5	3	1.0
M stage				
M0	718	86.7	256	84.9
M1	102	12.3	47	15.1
MX	8	1	0	0.0
Type of surgery				
Palliative	568	68.6	235	75.3
Radical	218	26.3	71	22.8
NOS	42	5.1	6	1.9
Lymph node dissection				
1–3 removed	65	7.9	21	6.7
≥ 4 removed	271	32.7	89	28.5
None/unknown	492	59.4	202	64.7

NOS, not otherwise specified.

The majority of patients were white (92.5%), male (77.3%), and had right‐sided lesions (61.4%). Based on the American Joint Committee on Cancer staging system, 187 stage I cases (16.4%), 204 stage II cases (17.89%), 358 stage III cases (31.40%), and 391 stage IV cases (34.30%) were enrolled. Because > 60% of the patients were stage III–IV, most surgical procedures were palliative.

In total, 577 patients had epithelioid histology (50.6%), 289 had sarcomatoid histology (25.4%), 168 had biphasic histology (14.7%), and 107 had fibrous histology (9.4%). Because the differentiation of resected tumors was not regularly recorded in the SEER database, most patient differentiations were NOS. Of these patients, 686 (60.18%) were administered cytotoxic chemotherapy and 285 (25.0%) were administered radiotherapy. Among the entire cohort, median OS was 14 months (95% CI 13–15 months). The one, three, and five‐year OS rates were 53.9%, 17.1%, and 8.5%, respectively.

### Predictors of overall survival and model specifications

We first selected 14 clinically relevant candidate variables from the database: age at diagnosis, race, gender, differentiation, histology, radiotherapy, chemotherapy, primary tumor location, clinical stage, tumor node metastasis (TNM) stage, surgery type, and lymph node dissection. In univariate analysis, the factors significantly associated with reduced OS were: advanced age, male gender, fibrous histology, poor differentiation, undifferentiated, and treatment without chemotherapy or radiotherapy (Table 2). Backward stepwise selection using the AIC in Cox proportional hazards regression modeling identified eight variables that were included in the final model. Table 2 presents the HRs and 95% CIs for the multivariate Cox proportional hazards regression analysis for variables selected by the AIC. Gender (HR 1.486, 95% CI 1.241–1.779), differentiation (HR 2.312, 95% CI 1.362–3.921), histology (HR 1.478, 95% CI 1.088–2.005), lymph node metastasis (HR 1.409, 95% CI 1.155–1.718), treatment without chemotherapy (HR 1.319, 95% CI 1.130–1.538), and lymph node dissection (HR 1.350, 95% CI 1.025–1.776) were each independently associated with OS (all *P* < 0.05), while the surgery type (HR 1.234, 95% CI 0.868–1.753) tended to be associated with prognosis.

**Table 2:** Factor and overall survival associations via the Cox proportional hazards regression model in the training cohort (*n* = 828)

	Univariable analysis			Multivariable analysis		
Prognostic factor	HR	95% CI	*P*	HR	95% CI	*P*
Factors selected						
Age	1.004	0.992–1.017	0.500	1.003	0.990–1.017	0.600
Age’†	1.030	1.014–1.047	< 0.001	1.027	1.009–1.044	0.002
Gender						
Female	Ref	—	—	Ref	—	—
Male	1.427	1.196–1.703	< 0.001	1.486	1.241–1.779	< 0.001
Histology						
Biphasic	Ref	—	—	Ref	—	—
Epithelioid	0.689	0.554–0.859	< 0.001	0.682	0.543–0.855	< 0.001
Fibrous	1.675	1.244–2.254	< 0.001	1.478	1.088–2.005	0.012
Mesothelioma	0.878	0.693–1.113	0.283	0.734	0.574–0.939	0.014
Differentiation						
Well or moderately	Ref	—	—	Ref	—	—
Poorly	2.463	1.469–4.131	< 0.001	2.312	1.362–3.921	0.002
Undifferentiated	2.228	1.147–4.328	0.018	1.468	0.738–2.916	0.273
NOS	1.868	1.209–2.887	0.005	1.735	1.113–2.704	0.015
Chemotherapy						
Yes	Ref	—	—	Ref	—	—
No	1.350	1.168–1.561	< 0.001	1.319	1.130–1.538	< 0.001
N stage						
N0	Ref	—	—	Ref	—	—
N1	0.950	0.766–1.178	0.640	1.342	1.068–1.685	0.012
N2	1.122	0.928–1.357	0.235	1.409	1.155–1.718	< 0.001
N3	1.333	0.732–2.426	0.348	1.480	0.809–2.707	0.203
NX	1.356	0.884–2.080	0.162	1.006	0.647–1.561	0.980
Lymph node dissection						
1–3 removed	Ref	—	—	Ref	—	—
≥ 4 removed	0.880	0.661–1.171	0.380	0.968	0.722–1.296	0.827
None/unknown	1.382	1.053–1.814	0.019	1.350	1.025–1.776	0.032
Type of surgery						
NOS	Ref	—	—	Ref	—	—
Palliative	1.093	0.789–1.514	0.592	1.234	0.868–1.753	0.240
Radical	0.819	0.581–1.155	0.255	1.077	0.739–1.572	0.698
Factors not selected						
Race						
White	Ref	—	—			
Black	0.981	0.672–1.431	0.920			
Other	0.689	0.458–1.035	0.073			
Radiotherapy						
Yes	Ref	—	—			
No	1.278	1.088–1.501	0.003			
Primary tumor location						
Bilateral	Ref	—	—			
Left‐sided	1.171	0.657–2.087	0.593			
Right‐sided	1.326	0.748–2.353	0.334			
Clinical stage						
I	Ref	—	—			
II	0.988	0.772–1.265	0.924			
III	0.920	0.737–1.148	0.460			
IV	1.202	0.966–1.467	0.099			
T stage						
T1	Ref	—	—			
T2	1.056	0.855–1.305	0.611			
T3	0.930	0.752–1.151	0.503			
T4	1.177	0.953–1.454	0.130			
TX	1.085	0.402–2.930	0.873			
M stage						
M0	Ref	—	—			
M1	1.191	0.958–1.480	0.115			
MX	1.512	0.717–3.188	0.277			

† Age’ is constructed as a spline variable (when k = 3). A model selection technique based on the Akaike information criteria was used. NOS, not otherwise specified.

**Figure 2:**
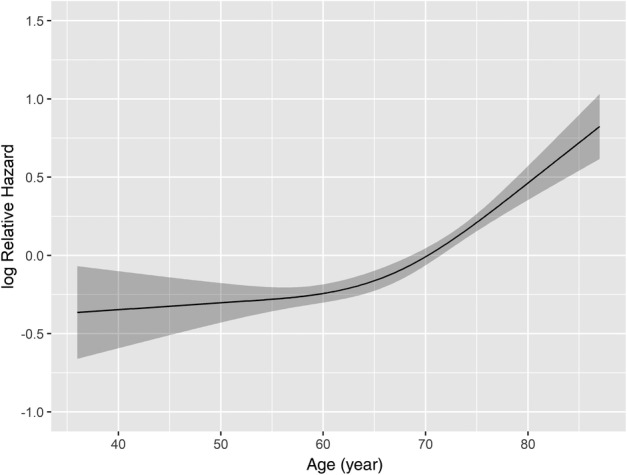
Continuous variable transformation in univariate analysis via restricted cubic splines concerning age.

To construct the final model, we used restricted cubic splines to examine the continuous variable effects. The variable of age at diagnosis had nonlinear effects on the log of the hazard ratio of OS (Fig 2); age was thus optimally modeled with three knots, with the extremes showing the highest mortality risk.

## Nomogram

**Figure 3:**
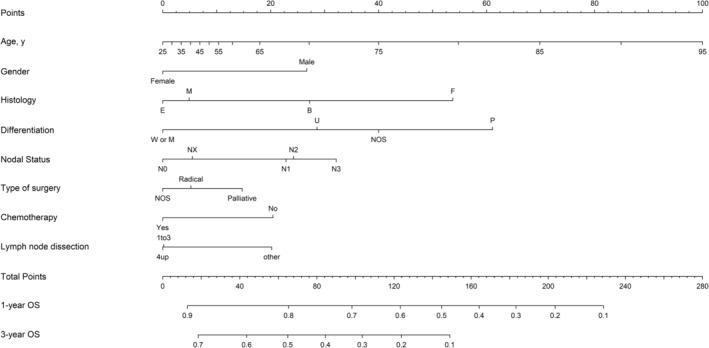
Prediction of overall survival (OS) of patients who underwent surgery according to the nomogram. Histology: B, biphasic mesothelioma; E, epithelioid mesothelioma; F, fibrous mesothelioma; M, mesothelioma. Differentiation: M, moderately differentiated; P, poorly differentiated; NOS, not otherwise specified; U, undifferentiated; W, well differentiated.

**Figure 4:**
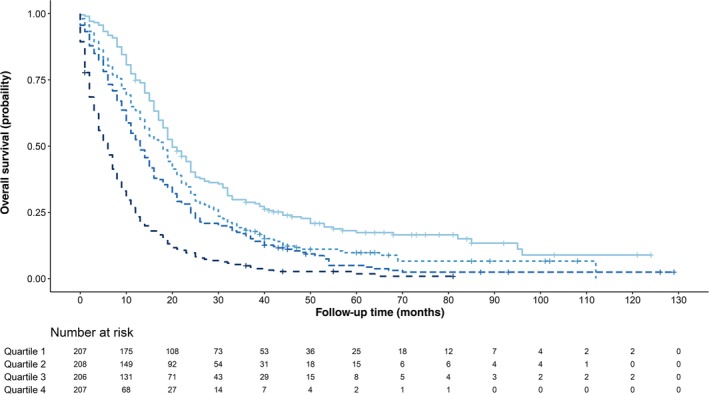
Overall survival of patients who underwent surgery based on the quartiles of the nomogram predicted score. 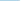 *Quartile 1*, 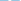 *Quartile 2*, 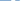 *Quartile 3*, 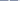 *Quartile 4*.

A nomogram model was created to predict the OS of MPM patients who underwent surgery (Fig 3). A high score in the nomogram was associated with a poor prognosis. We divided the predicted nomogram scores into quartiles and plotted their survival curves (Fig 4). The nomogram was able to stratify patients into four distinct prognostic groups (quartile 1: 1‐year survival rate, 74.9%; quartile 2: 1‐year survival rate, 63.5%; quartile 3: 1‐year survival rate, 52.4%; and quartile 4: 1‐year survival rate, 13.3%; *P* < 0.001).

### Model performance and validation in the training cohort

**Figure 5:**
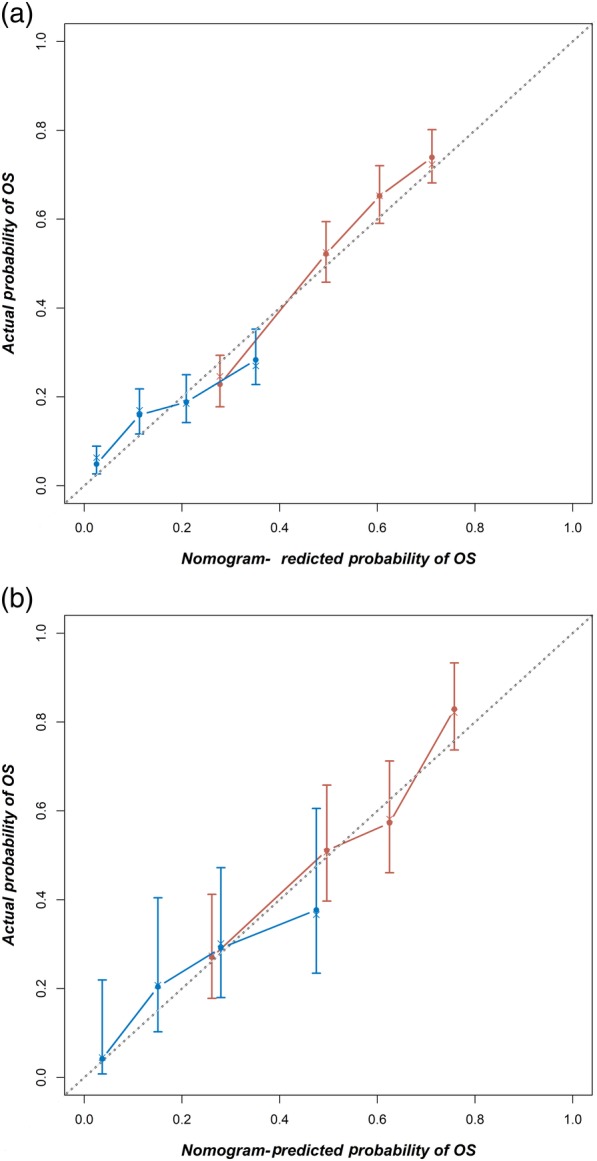
Calibration curves to predict overall survival (OS) in the (**a**) training, 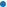 1‐year OS, 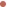 3‐year OS and (**b**) validation cohorts, 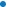 1‐year OS, 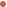 3‐year OS. OS predicted by the nomogram is plotted on the *x*‐axis, while the actual probability of OS is on the *y*‐axis. A 45‐degree curve (dotted line) would mean that the model was perfectly calibrated such that the predicted probabilities and actual outcomes were identical.

In the training cohort, the Harrell's C‐index for the established nomogram to predict OS (0.715, 95% CI 0.698–0.736) was significantly higher than that of the TNM staging system (0.564, 95% CI 0.539–0.589; *P* < 0.01). A calibration plot showed high consistency between predicted and actual survival in the training cohort at one and three years (Fig 5a).

We further tested the nomogram with a validation cohort (*n* = 312). The C‐index was significantly higher in the nomogram (0.656, 95% CI 0.645–0.667) than in the TNM system (0.543, 95% CI 0.532–0.554; *P* < 0.01). A calibration plot for the nomogram for predicting one‐year and three‐year survival indicated that the nomogram has high predictive accuracy in the validation cohort (Fig 5b).

## Discussion

To our knowledge, this was the first large‐scale study to analyze the prognosis of MPM patients who underwent surgery using a nomogram based on data from the SEER database. In a training cohort of 828 cases, independent prognostic factors were identified, including age, gender, histology type, differentiation, N stage, chemotherapy, type of surgery, and lymph node dissection. We developed a nomogram that could enable us to visually evaluate patient survival. An advantage of this study was the use of a validation cohort that consisted of 312 patients to evaluate the performance of the nomogram, which makes our conclusions more convincing. The nomogram showed good discriminative ability and was well calibrated. These results improve our understanding of resected MPM lesions and provide a reliable tool for predicting patient OS.

There have been several studies of survival involving a SEER dataset of MPM patients.^8^, ^11^, ^18^, ^19^, ^20^, ^21^, ^22^ Taioli *et al.* reported median survival of 6.5 months in patients who were not administered radiotherapy or surgery, 14.5 months in the surgical group, and 13 months in patients administered both radiation and surgery.^22^ These data are similar to our finding of 14 months median OS, thus supporting our opinion that the prognosis of surgically treated MPM patients should be analyzed separately from non‐surgery patients. In a study by Yang *et al.* based on a SEER dataset, the survival rate in surgically treated patients was higher than that in our data (1‐year survival 63% vs. 53.9%; 3‐year survival 21% vs. 17.1%).^11^ This may be because Yang *et al.* excluded patients with sarcomatoid histology and stage IV disease. In general, the survival data in our study are comparable to data presented in earlier reports.

Similar to our findings, prior studies have shown that female gender, younger age, and early stage are independent predictors of longer survival in multivariate analysis.^8^, ^22^ It is interesting that several studies have found that female MPM patients have a better prognosis than male patients^21^, ^22^ this would appear to be worth investigating further. In contrast to the study by Wang *et al.*,^8^ the diagnoses in our patient cohorts were based on resected surgical specimens while cases involving only a small biopsy were excluded, which enhances the accuracy of our diagnoses and the reliability of pathological staging compared to clinical staging in survival analysis.

In accordance with previous reports, we also found that advanced age was a significant factor associated with poor survival.^3^, ^11^, ^19^ Furthermore, we found that the age at diagnosis had nonlinear effects on the log of the hazard ratio of OS, which has not previously been reported in MPM. In a Cox proportional hazards survival model constructed by Yang *et al.*, surgery was associated with improved survival in patients aged ≥ 70 years but not in those aged ≥ 80.^11^ Consistent with this result, our nomogram also showed that the mortality risk rises sharply with the increase in age in patients aged > 70. These data suggest that we must be very cautious in deciding whether or not to perform surgery on patients aged > 70 years, as it may not translate into a survival benefit.

We also found that survival is significantly affected by tumor histology. The biphasic subtype showed poor prognosis compared to the epithelioid subtype, whereas the sarcomatoid subtype was even poorer. This finding is consistent with prior studies.^19^, ^23^, ^24^, ^25^, ^26^, ^27^ Meyerhoff *et al.* reported that epithelioid histology is related to better survival compared to sarcomatoid and biphasic histology.^19^ Surgery significantly improves survival for epithelioid MPM patients, but sarcomatoid and biphasic patients have poor prognoses and may not benefit from surgery. The specific histologic type should be considered during the surgical decision‐making process. In addition, our data showed that tumor differentiation is associated with survival in MPM patients. Well or moderately differentiated subtypes resulted in better prognoses than poorly differentiated or undifferentiated subtypes. However, in our cohort, most MPM tumors were listed as NOS, indicating the importance of determining the differentiation status in order to make a pathological diagnosis of MPM.

It has been reported that TNM staging is related to MPM prognosis;^28^, ^29^, ^30^, ^31^ however, we found no significant association between OS and TNM staging. Similarly, Meyerhoff *et al.* observed no survival difference between different clinical stages within the epithelioid histotype.^19^ In the IASLC‐IMIG study, Rusch *et al.* also reported no significant differences in survival among the patients of different stages that underwent surgery.^12^ One possible reason for this is that most patients in this study (70.4%) underwent palliative rather than curative surgery. More studies are needed to resolve this issue.

Regarding the heterogeneity within our patient population, optimal management suggests surgery within multimodality therapy to suitable patients, for example, young patients with early‐stage epithelioid histology. As the most common site of recurrence after surgery is the ipsilateral hemithorax, involvement of the N1 and N2 stations poses an increased risk,^10^, ^13^ thus future postoperative treatment should consider improved radiotherapy techniques, as well as new target and immunotherapy strategies.^32^, ^33^

This study has several limitations. First, because it is retrospective, large randomized controlled trials are needed to confirm our findings. Second, many other factors can influence the outcome of surgery, including cardiopulmonary conditions and comorbidities. However, this information is not available in the SEER database. Furthermore, detailed information of the chemotherapy regimens and radiation doses was not available in the SEER database; therefore, we could not further analyze the association between OS and these factors.

More research is needed to improve the nomogram so that it will better predict patient survival. Identification of the key factors associated with survival enables us to plan individualized treatments that will provide the most benefit to patients.

## Disclosure

No authors report any conflict of interest.
